# Assessing the effect of *Aedes* (*Stegomyia*) *aegypti* (Linnaeus, 1762) control based on machine learning for predicting the spatiotemporal distribution of eggs in ovitraps

**DOI:** 10.1016/j.dialog.2022.100003

**Published:** 2022-02-09

**Authors:** Rafael Piovezan, Thiago Salomão de Azevedo, Euler Faria, Rosana Veroneze, Claudio José Von Zuben, Fernando José Von Zuben, Maria Anice Mureb Sallum

**Affiliations:** a Universidade de São Paulo, Faculdade de Saúde Pública, Departamento de Epidemiologia, São Paulo, SP, Brazil; b Universidade Estadual Paulista, Departamento de Zoologia, Rio Claro, SP, Brazil; c Universidade Estadual de Campinas, Faculdade de Engenharia Elétrica e de Computação, Campinas, SP, Brazil

**Keywords:** *Aedes aegypti*, Surveillance, control, Machine Learning, dengue

## Abstract

**Background:**

*Aedes aegypti* is the dominant vector of several arboviruses that threaten urban populations in tropical and subtropical countries. Because of the climate changes and the spread of the disease worldwide, the population at risk of acquiring the disease is increasing.

**Methods:**

This study investigated the impact of the larval habitats control (CC), nebulization (NEB), and both methods (CC + NEB) using the distribution of Ae. aegypti eggs collected in urban area of Santa Bárbara d'Oeste, São Paulo State, Brazil. A total of 142,469 eggs were collected from 2014 to 2017. To verify the effects of control interventions, a spatial trend, and a predictive machine learning modeling analytical approaches were adopted.

**Results:**

The spatial analysis revealed sites with the highest probability of Ae. aegypti occurrence and the machine learning generated an asymmetric histogram for predicting the presence of the mosquito. Results of analyses showed that CC, NEB, and CC + NEB control methods had a negative impact on the number of eggs collected in ovitraps, with effects on the distribution of eggs in the three weeks following the treatments, according to the predictive machine learning modeling.

**Conclusions:**

The vector control interventions are essential to decrease both occurrence of the mosquito vectors and urban arboviruses. The inference processes proposed in this study revealed the relative causal impact of distinct mosquito control interventions. The spatio-temporal and the machine learning analysis are relevant and Powered by Editorial Manager® and ProduXion Manager® from Aries Systems Corporation robust analytical approach to be employed in surveillance and monitoring the results of public health programs focused on combating urban arboviruses.

## Highlights

• Controlling *Aedes aegypti* has been one of the greatest challenges worldwide in the last decades.
• In the Americas alone, an estimated 11,740 million people have been infected with the dengue virus in the last six years.
• The use of the machine learning analytical approach is an innovation in monitoring mosquito control action.
• The innovative approach can better guide policies to fight arboviruses transmitted by *Ae. aegypti*.

## Graphical abstract

**Figure:**
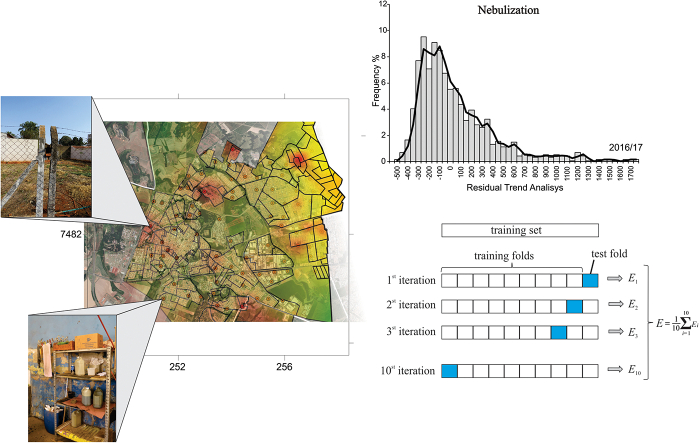

## 1 Introduction

It is estimated that about 2.5 billion people are at risk of acquiring Dengue virus infections worldwide [1]. In addition to Dengue, mosquitoes can carry almost 4000 arboviruses [2] including Mayaro, Venezuelan equine encephalitis, Eastern equine encephalitis, Chikungunya, yellow fever, Rocio, Saint Louis encephalitis, West Nile, and Oropouche [3]. Malaria and bancroftian filariasis are other mosquito-borne diseases that affect populations inhabiting tropical and subtropical areas [4].

*Aedes* (*Stegomyia*) *aegypti* (Linnaeus, 1762) is one of the most important mosquitos that threatens public health because it can adapt to the urban environment and potentially transmit arboviruses [5,6]. The habitats used by this species are usually anthropical [[7], [8], [9]]. The species is associated with several man-made habitats in urban areas, and vector control programs are exceedingly complex—especially in developing countries, which makes it difficult to control the species.

Dengue has recently received increasing attention worldwide and has infected more than 11,740 million people in the Americas approximately 6000 deaths in the last six years [10]. However, it is believed that the number of cases is underestimated and that there may be as many as 390 million cases annually [[11], [12], [13], [14]]. While most Dengue infections only cause mild symptoms such as fever, headache, myalgia, and arthralgia, a small percentage of people develop severe disease [15].

In Brazil, four Dengue serotypes are transmitted by *Ae. aegypti* [15]. The countrywide presence of this mosquito where several arboviruses circulate are a major threat to public health [16]. From 2015 to the 23rd epidemiological week in 2021, more than 6.618 million new Dengue cases and 3583 new deaths were reported [[17], [18], [19], [20], [21], [22], [23], [24]]. In addition, Brazil is currently impacted by other emerging arboviruses transmitted by *Ae. aegypti*. From 2015 to 2021, the chikungunya fever affected 845,958 people, and the Zika virus fever reached 262,104 cases [[17], [18], [19], [20], [21], [22], [23]]. The Zika virus is associated with microcephaly outbreak caused by a that is transmitted from pregnant women to their fetuses [25]. From the beginning of the epidemic in the 45 epidemiological weeks of 2015 to 2021, 3577 cases of congenital syndrome and microcephaly were noted in Brazil [26].

The development of improved vector control management in urban areas is essential to reduce the risk of epidemics and control transmission of arboviruses. A better understanding of the factors that contribute to the spread of Dengue in urban areas is needed for the development and prediction of more efficient management and vector control practices. Large investments have been made in the control of Dengue: Brazil spent more than USD $300 million in controlling *Ae. aegypti* in 2015 [27].

The control of *Ae. aegypti* encompasses three major approaches: vector surveillance and control, epidemiological surveillance of arboviruses suspected and confirmed cases, and robust social communication and health education programs [16,28]. The implementation of an effective vector control program depends on the government investments. For this reason, methods that allow one to optimize control and direct interventions to those locations of increased epidemiological risk are critical [12,[29], [30], [31]]. Therefore, effective control of *Ae. aegypti* will result in decreasing Dengue, Zika, and chikungunya infections in the urban populations exposed to the mosquito vectors [7,32].

The use of oviposition traps for surveillance of *Ae. aegypti* is a sensitive and safe way to help mosquito control [33,34]. Likewise, measuring the impact of interventions is of fundamental importance for program administrators. Based on this information, one can delineate measures and plans to adopt the most advantageous vector control methods. Thus, our study will evaluate the effectiveness of entomological surveillance and control measures delineated to eliminate *Ae. aegypti*. We investigated the results of interventions adopted based on the ovitraps findings using spatio-temporal analysis and predictive models considering field-collected data.

## 2 Materials and methods

### 2.1 Study area

**Fig. 1:**
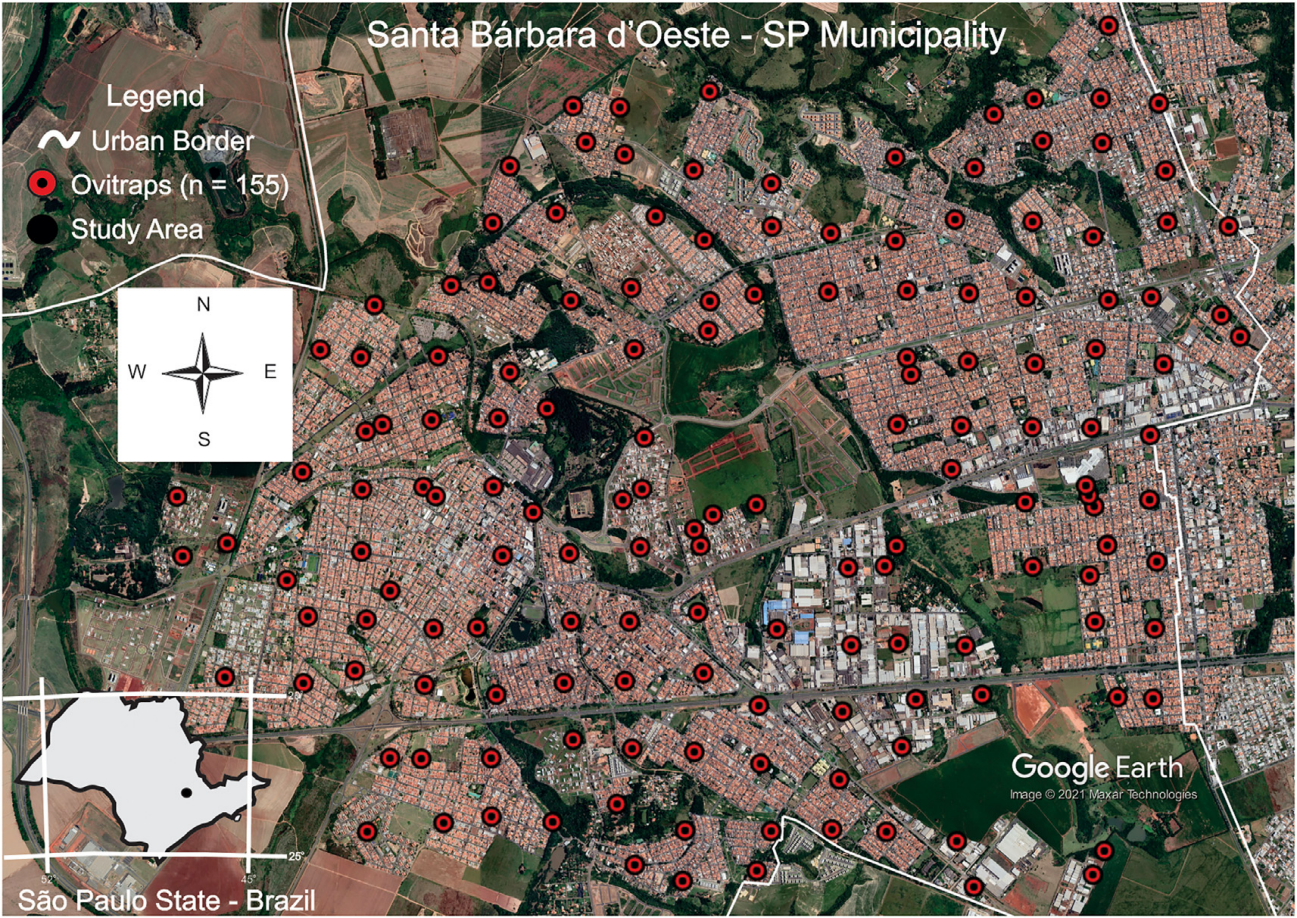
Study area and ovitraps: *n* = 155 - *Aedes aegypti -* (Brazil; São Paulo state and municipality Santa Bárbara d'Oeste, 2014 to February of 2017).

The field collections were carried out in the municipality of Santa Bárbara d'Oeste São Paulo state, southeastern Brazil (Fig. 1) (22.75° S; 49.38° W) at 560 m altitude. The total area of the municipality is 271 km^2^. In accordance with Koeppen [35], the climate is Cwa and tropical with a rainy summer and a dry winter. The mean precipitation is 1466.1 mm annually [36,37].

The investigation was based on egg collection using ovitraps installed throughout the city. The collection approach is described in Dengue control guides [16,28,38]. Larval and adult control interventions were included in the analyses. These interventions were larval habitat control (CC) and Blocking Nebulization (NEB). Both technologies, CC and NEB, were employed isolated (CC or NEB) and combined (CC + NEB) in each area of the respective trap.

### 2.2 Ovitraps

We distributed 155 ovitraps in the urban area of Santa Barbara d'Oeste. Egg collections employed standard methods and were routinely used for monitoring *Ae. aegypti*. The ovitraps were black pots with an approximate volume of one liter and 14 cm long, 3.5 cm wide and 0.5 cm thick wooden pallet. The distribution of traps was defined by superimposing a grid with quadrants on the map of the municipality with a spatial correlation among larval habitats of approximately 500 m (a radius of approximately 250 m) between the traps. This value is based on autocorrelation test of *Ae. aegypti* habitats and an exponential isotropic semi-variogram that demonstrated spatial correlation between habitats. There was no significant relationship beyond this distance. Importantly, this value is close to the values recommended for the control of larval habitats when Dengue cases are confirmed. This procedure is consistent with the importance of spatial distribution data for vector control and the delineation of transmission risk areas [9]. The centroid was determined in each quadrant, and the buildings were chosen randomly. Ovitraps (*n* = 155) were distributed in the urban area (one per quadrant) of the municipality and surveyed 52 weeks per year. The ovitraps were installed around the buildings from ground level to approximately 1.5 m high.

Field collections were done from 2014 to 2017. The traps were inspected by mosquito control personnel—pallets were removed and replaced by new pallets with the same dimensions and materials. The water levels were replaced in each ovitrap, and a new collection was performed in the following week. After removing the pallets, they were kept in plastic boxes to avoid damage and drying. In the laboratory, the eggs found on each pallet were counted under a stereomicroscope, and data on the number of eggs in each pallet were tabulated. The eggs were systematically removed from the pallets via brushes and disposed in chloride solution before washing in a sewage network. *Aedes* (*Stegomyia*) *albopictus* (Skuse, 1894) can occasionally lay eggs in the ovitraps. However, *Ae. aegypti* becomes the dominant species in these traps in highly urbanized landscapes [9].

### 2.3 Vector control

Larval habitats control and adult elimination using insecticide nebulization as primary activities were performed in Santa Barbara d'Oeste, State of São Paulo, Brazil. Larval habitat control included both active search and removal of any objects that can accumulate water and favor the development of the immature stages of *Ae. Aegypti*: This species is known to use domestic anthropic breeding sites [[7], [8], [9]]. Collections were carried out in houses visited by vector control personnel. This activity aimed to study the control of immature mosquito stages by eliminating the oviposition habitats.

Adult control used insecticide nebulization. It was employed to block the transmission of Dengue virus because the intervention is primarily focused on eliminating potentially infected females from areas with active transmission. Nebulization for the control of adult mosquitoes requires spraying with organophosphate insecticide (Malathion GT 96%) using light or heavy equipment, e.g., ultra-low volume (ULV)-dispersing micro drops with diameters of 5 and 30 μm [16]. All control interventions are described in Dengue control guidebooks [16,28,39]. The vector control activities were performed according to the same procedure in different areas of the municipality, by the same control personnel of the municipality and with the same training and technical skills.

The control actions (CC, NEB, and CC + NEB) were performed in the ovitraps as a function of the demand of notifications of suspected and positive cases of Dengue. For each ovitrap selected through the mathematical model, an area with a radius of 250 m received one type of treatment to stop Dengue transmission. The control in each area was performed once during the follow-up period of the respective trap. Thus, treatment was performed in accordance with the occurrence of Dengue cases.

## 3 Calculations

### 3.1 Spatial analysis

The ovitraps were georeferenced and plotted in the Santa Bárbara d'Oeste municipality map. Spatial and temporal analyses were performed from 2014 until 2017. The field data was organized into three periods: (1) 2014; (2) 2015, and (3) 2016 to February of 2017. The total number of eggs collected in each trap yearly were summarized. This statistical analysis was done to spatially verify the efficiency of the control methods employed, i.e., places with different types of treatments have different numbers of eggs.

A first-degree trend analysis was performed to transform the database into an image using a grid of the estimated number of eggs found in the traps. This mapping technique consisted of a method in which a continuous surface is fitted to *Z* values as a linear function of the XY coordinates of the irregularly distributed sampled points. For this, we adopted a first-degree polynomial equation with multiple regression between the values of the attribute investigated and the geographic locations [40,41].

The equation used was:

(1) $$Z_{i}=a+{\mathrm{bx}}_{i}+{\mathrm{cy}}_{i}$$

where *Z*_*i*_ corresponds to the predicted value of the number of eggs at trap *i* and *x*_*i*_ and *y*_*i*_ are the corresponding geographic coordinates.

This analysis was carried out using Eq. (1) because it allowed us to visualize the local fluctuations represented by the residual values. These deviations were obtained by subtracting the surface of the original data from the first-degree trend, thus resulting in a surface of deviations (residuals). The residuals can be positive when the original surface values are greater than those interpolated on the trend surface or negative when the situation is opposite. The residuals were used to observe the areas where there was a tendency to increase or decrease the number of eggs for each year of field collection. Values with the point deviations from the calculated surface were found via the dependent variable in relation to the predicted value via a regression line according to the following Eq. [41]:

(2) $$\lambda_{\mathrm{ii}}=Z_{i}^{*}-Z_{i}$$

where *Z*_*i*_∗ corresponds to the real number of eggs in trap *i*.

This spatial analysis technique allows one to investigate intercalary relationships of the same spatial variable, thus confronting regional trends with local anomalies. Following this round of analysis, the annual quantity fluctuation maps of *Ae. aegypti* eggs were compiled from 2014 to 2017 employing data interpolation via the minimum curvature algorithm. This method was used because it smoothed the information, thus providing a cartographic result that was more representative of the original data [41].

In the final round of analysis, a database on the location of the blocks where the insecticide nebulization and larval control were applied were georeferenced and overlaid on the residual maps of surface trend. We then extracted values of local fluctuations in the number of eggs studied per year and the interventions adopted to control *Ae. aegypti* habitats. Six graphs were prepared to show the behavior of the anomalies of the occurrence (local deviation surface) of *Ae. aegypti* eggs associated with each control method adopted.

### 3.2 Mathematical modeling

The innovative use of machine learning seen here allowed one to predict those traps with a promising time interval free of *Ae. aegypti* eggs. These regions were a radius of 250 m from the ovitraps installed in the centroids of the quadrants. In this area, the traps that presented some type of treatment (CC, NEB or CC + NEB) within this six-week interval were defined as having control action between the first three and the last three weeks. This interval of three weeks before the control was established was integrated so that the predictive model would have robust conditions to estimate the number of eggs collected in the following three weeks if there was no control intervention. Our results uncovered 185 promising regions. The details of the machine learning approach are presented below as well as in the Supplementary Material.

#### 3.2.1 Inferring the effect of a control event using the causal impact

An accurate estimation of the impact caused by an intervention on any spatio-temporal process of interest is quite relevant for strategic decision-making [42]. Here, we should compare two basic scenarios: (1) the real scenario of the process evolution after intervention; and (2) the hypothetical (unobserved) scenario of what would have happened in the process evolution without an intervention. Given a high confidence estimation of the hypothetical scenario 2, it is possible to measure the causal impact of the intervention, thus properly characterizing the cost/benefit relation of the intervention. Though several approaches have already been proposed to perform causal inference [43], the approach described here is inspired by the methodology supporting the Causal Impact software package proposed by Brodersen and colleagues [44] where the time series associated with the two scenarios just described (a real and a synthetic time series) are compared. The synthetic time series is obtained here via a machine learning approach responsible for training a nonlinear learning model from available data, thus guiding a high-performance time series prediction of the occurrence of eggs in ovitraps when no intervention is considered.

#### 3.2.2 Data processing

This section is divided into three subsections: First, crude datasets explain all data preparation procedures conceived to build a single time coherent dataset for feeding further analyses. Second, training datasets explain the procedures conceived for getting examples to train three machine learning models required for applying the causal impact approach. Finally, the inference dataset was obtained to explain the procedures that were developed to acquire samples of distinct scenarios of the control strategies.

#### 3.2.3 Raw datasets

Three datasets were made available: (1) collection of eggs, (2) control events, and (3) rainfall index with data ranging from January 2014 to February 2017. All information was manually annotated by the health professionals of Santa Barbara d'Oeste. There was no key (unique row identifier) to join the information of these three datasets into a single dataset that could describe the time series of all variables of interest for each location. Therefore, a series of procedures was developed to produce a single dataset for later analyses: The collection of eggs dataset is composed of a vast number of discrete events with non-constant time intervals. This characteristic is due to the sporadic pattern of egg collection. An example of this dataset is presented in Table 1 of the Supplementary material.

Nonetheless, a full history of these events can be sampled from the dataset for each location. We pre-processed this data to achieve this goal by producing a weekly time series for each location in the city while considering the total number of *Ae. aegypti* eggs collected for that week and location. Any week for a specific location that did not have any larval counting was flagged as −1 in further analyses (representing a missing value). Table 2 of the Supplementary material displays an example of the egg collection dataset after treatment.

The daily rainfall index dataset was used to calculate the average and standard deviation of the rainfall index for each week of the year. Table 3 of the Supplementary material presents an example of the rainfall index dataset after treatment.

The original dataset of control interventions has a similar pattern as the original egg collection dataset. Both are composed of discrete events with the weekly collection of ovitraps. The control intervention has a non-constant time interval resulting from the epidemiological concepts presented in point 2.3. The characteristics of each type of control strategy (NEB, and CC) are described above. Routines to build a weekly time series for each location were developed. Table 4 of the Supplementary Material presents an example of the control intervention dataset after treatment.

After each of the three original datasets was pre-processed in isolation, the resulting information was compiled into a single dataset using the year, week, and location. The final dataset for the 1976 locations from 2014 to February of 2017 resulted in 322,088 rows and 8 columns. Table 5 of the Supplementary Material presents an example of this final dataset.

#### 3.2.4 Training dataset

After producing a single dataset with all necessary information, a routine was developed to find space-time promising regions with the following requirements:

• Time: 6-week time window.
• Region: 250-m radius around each location (i.e., pair of latitude and longitude) available in the dataset.
• No intervention: only time windows in which a control strategy intervention did not take place were considered.

This routine aimed to find promising spatio-temporal regions displaying the evolution pattern of the *Ae. aegypti* eggs over time in normal conditions. This routine yielded 1748 samples. Fig. 1 of the Supplementary Material shows an example of a space-time promising region.

These samples were used to train three non-linear autoregressive units with exogenous entries (NARX) machine learning models to forecast the number of *Ae. aegypti* eggs three weeks ahead: t + 1, t + 2, t + 3. The procedures used for training the models are described in the subsection on proposed models.

#### 3.2.5 Inference dataset

Samples of the intervention methods should be analyzed to measure their causal impact on the target variable. Therefore, we performed another search algorithm that could find samples in a six-week window with a control strategy intervention in the third week from regions composed of a radius of 250 m around each location. This search algorithm could identify 185 samples split into three scenarios:

• Scenario 1: 85 samples with only larval habitat control intervention in the third week.
• Scenario 2: 69 samples with larval habitat control and nebulization interventions in the third week.
• Scenario 3: 31 samples with only nebulization intervention in the third week.

Fig. 2 of the Supplementary Material shows an example of three samples from the inference dataset.

#### 3.2.6 Proposed models

We used three non-linear models that were autoregressive with exogenous entries to forecast the number of eggs collected in the next three weeks configuring a direct multi-step forecasting strategy. Each model was responsible for forecasting one week ahead, t + 1, t + 2, and t + 3. The machine learning model selected for addressing this task was the Extreme Gradient Boosting Machine (XGBoost) [45].

The predictors and the target variable assigned to each model is described in the list that follows.

• Model 1:

Predictors:

rainfall − mean(t − 3), rainfall − std.(t − 3), eggs(t − 3),

rainfall − mean(t − 2), rainfall − std.(t − 2), eggs(t − 2),

rainfall − mean(t − 1), rainfall − std.(t − 1), eggs(t − 1),

rainfall − mean(t), rainfall − std.(t).

Target: eggs(t + 1).

• Model 2:

Predictors:

rainfall − mean(t − 3), rainfall − std.(t − 3), eggs(t − 3),

rainfall − mean(t − 2), rainfall − std.(t − 2), eggs(t − 2),

rainfall − mean(t − 1), rainfall − std.(t − 1), eggs(t − 1),

rainfall − mean(t), rainfall − std.(t).

rainfall − mean(t + 1), rainfall − std.(t + 1).

Target: eggs(t + 2).

• Model 3:

Predictors:

rainfall − mean(t − 3), rainfall − std.(t − 3), eggs(t − 3),

rainfall − mean(t − 2), rainfall − std.(t − 2), eggs(t − 2),

rainfall − mean(t − 1), rainfall − std.(t − 1), eggs(t − 1),

rainfall − mean(t), rainfall − std.(t).

rainfall − mean(t + 1), rainfall − std.(t + 1).

rainfall − mean(t + 2), rainfall − std.(t + 2).

Target: eggs(t + 3).

All data (1,748 samples) was first normalized and then divided using a hold-out validation scheme with 80% (1,427 samples) for training and 20% (357 samples) for testing. The training set was used for training the models and optimizing their hyper-parameters using the methodology explained below. The test set was left untouched and was only used to evaluate the model performance.

Bayesian global search optimization (BSO) was used to select the model parameters [46]. The boundaries of each parameter (i.e., the maximum and minimum values) was configured to limit the search space. Table 1 presents the search space defined for the BSO process. Default values were used for all other parameters.

**Table 1:** Search space of parameters.

Parameter	Min	Max
subsample	0.7	1
min*c*_*hild*_*w*_*eight*_	1	20
reg*l*_*ambda*_	1*e*^*−*4^	10

The BSO aimed to find a local maximum (i.e., a minimum loss value) on the search space created by the distribution of values of each parameter respecting the minimum and maximum values configured.

A 10-fold cross-validation was performed at each BSO process iteration as shown in Fig. 3 in Supplementary Material.

The loss function selected for guiding the optimization process was the mean squared error (MSE) as presented in Eq. (3):

(3) $$E_{i}=\frac{1}{n}\sum_{j=1}^{n}{\left({\hat{y}}_{j}-y_{j}\right)}^{2}$$

Here, the index *j* represents a sample of the training fold ranging from 1 to *n*, *n* is the total number of samples in the training fold, ${\hat{y}}_{j}$ is the predicted value, *y*_*j*_ is the true observed value, and the index *i* is the iteration of the *k*-fold.

As a result, each iteration of the BSO is a complete run of the *k*-fold scheme on the training dataset with a final score *E* being the average MSE across all 10 folds (Eq. 4). The standard deviation of the error is presented in Eq. 5:

(4) $$E=\frac{1}{10}\sum_{i=1}^{10}E_{i}$$

(5) $$\sigma =\sqrt{\frac{\sum_{i=1}^{10}{\left(E_{i}-E\right)}^{2}}{10}}$$

The main goal of the BSO process was to find the best set of hyper-parameters for each model that yields a minimum value of *E*. In other words, it was desirable to minimize the average of the MSE across all 10 folds of the *k*-fold cross-validation.

## 4 Results

We collected 142,469 eggs in the ovitraps during field activities. The average number of eggs collected weekly in each trap was 13.9. The minimum and maximum number of eggs collected per trap was 0.0 and 357, respectively, with a variance of 51.3. Spatial analysis was used to correlate the effects of control events in space and time. The analytical approach employed a model that included the control interventions within a maximum radius of 250 m from each trap. The results of the spatial analyses and mathematical modeling are shown separately.

### 4.1 Spatial analysis

**Fig. 2:**
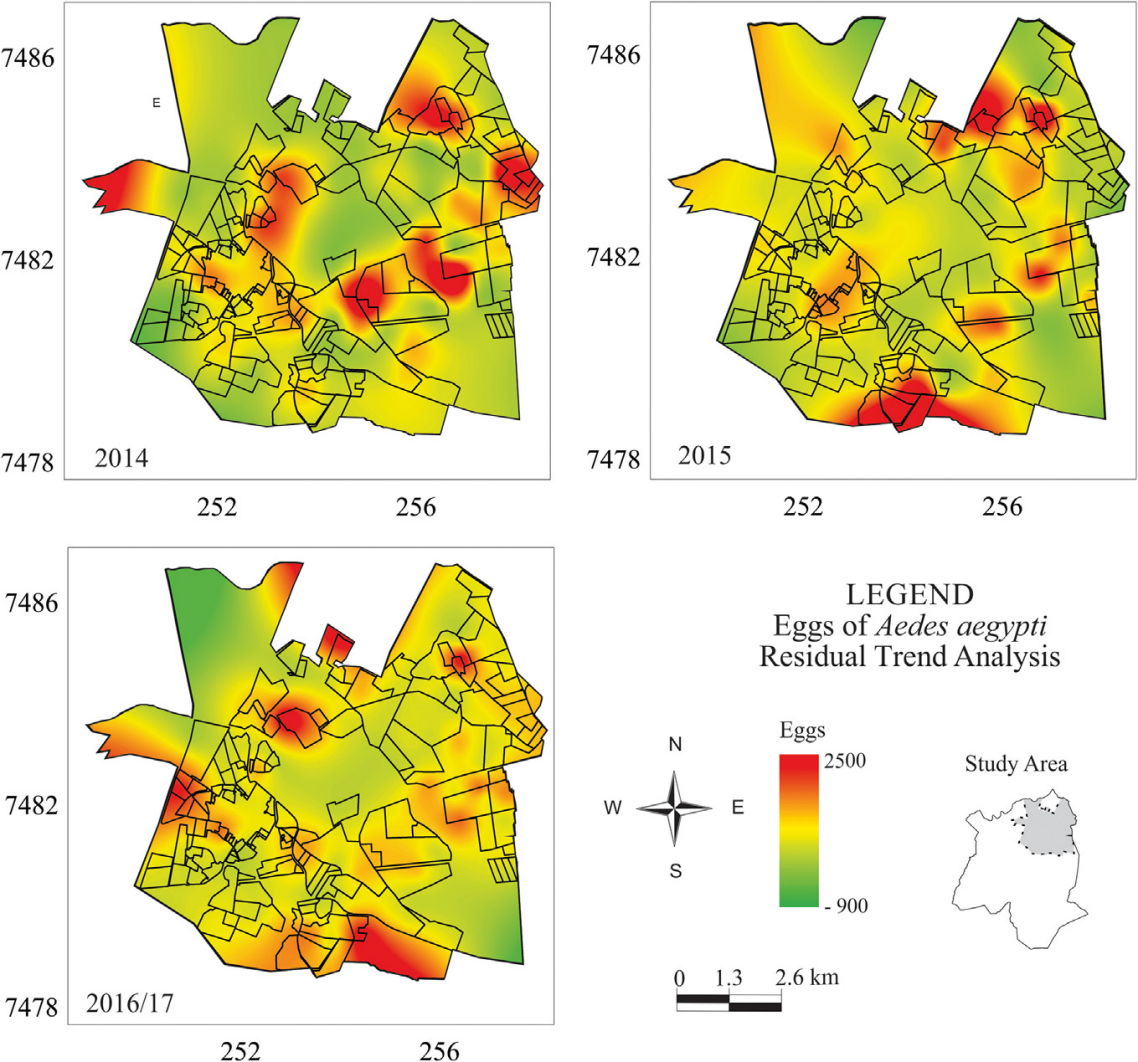
Residual trend maps of the distribution of eggs collected in ovitraps (2014; 2015; 2016 to February of 2017).

The spatial analysis generated trend residue maps for 2014, 2015, and 2016 to February of 2017 (Fig. 2). The maps show the points in the municipality where the trends of egg occurrence were above or below average, thus indicating where there was a greater concentration of eggs in the traps. The trend maps showed that the most critical points varied during the three periods studied. The variation correlated to the control interventions performed.

**Fig. 3:**
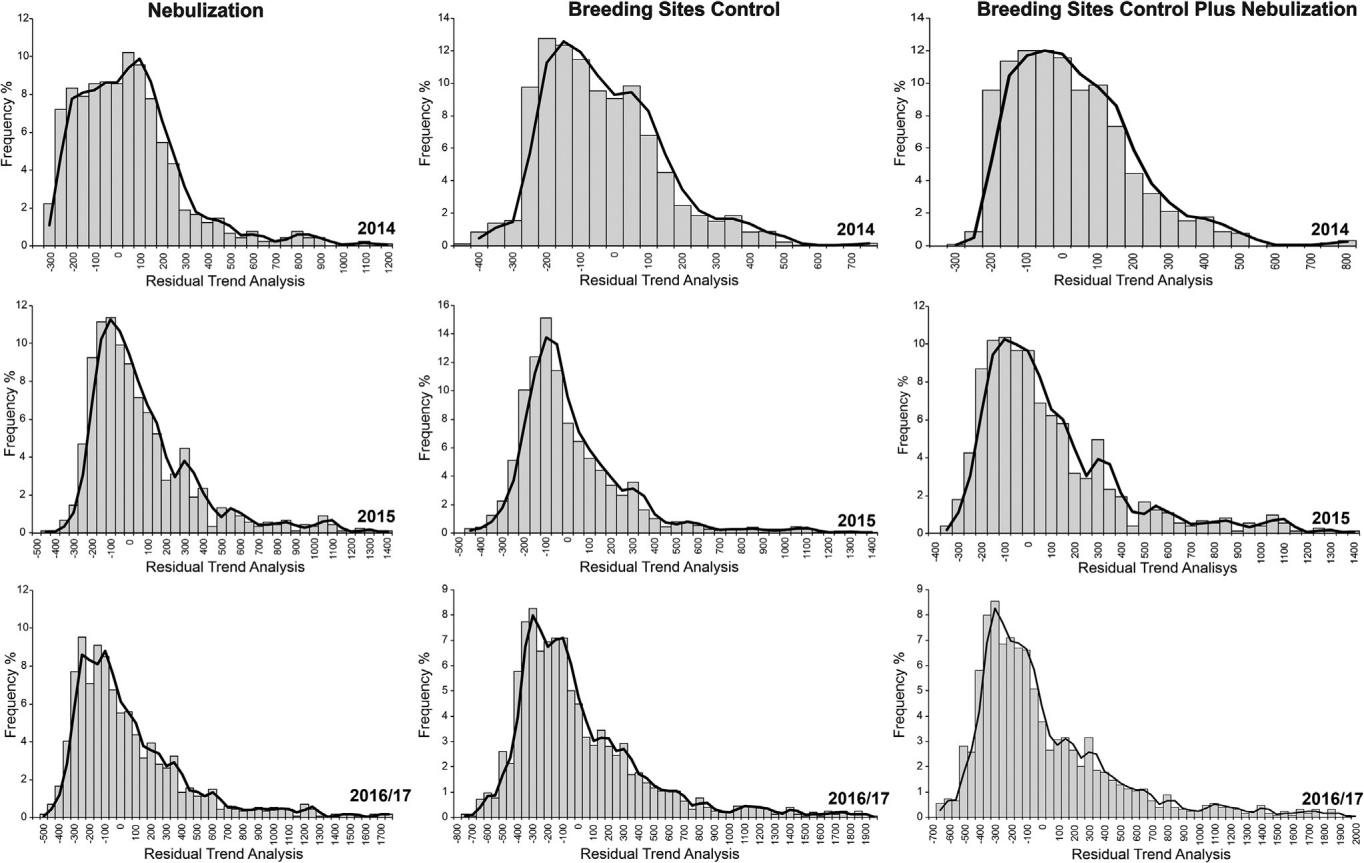
Histogram resulting from the correlation of trend residues from collections with ovitraps and the application of each treatment (CC; CC + NEB; NEB) in 2014, 2015 and 2016–2017.

The histograms generated by the correlation between the trend residue maps and the control actions (CC, NEB, CC + NEB) showed an asymmetric distribution that shifted to the left (Fig. 3). This result indicates that the trend in the number of eggs collected was lower in areas where vector control was performed than in areas that did not receive the treatments.

### 4.2 Mathematical modeling

Here, 85 CC events, 69 CC + NEB, and 31 NEB were included. For the modeling analysis, the assumptions described in the Methods section were applied. Table 2 shows the results of the modeling performance obtained at the best iteration of the BSO process for each of the three models (*t* *+* *1*, *t* *+* *2* and *t* *+* *3*) in terms of average error, *E*, and standard deviation of the error, *σ*. Table 2 also presents the error obtained for the test dataset.

**Table 2:** Results in the train and test dataset.

Model	*E*	*σ*	Test dataset
*t*_1_	0.0015	0.0008	0.0006
*t*_2_	0.0027	0.0009	0.0018
*t*_3_	0.0028	0.0007	0.0027

Table 2 shows the generalization capacity of the pattern learned during the training process. This group of models underscores the ability to forecast three weeks ahead the amount of mosquito eggs under normal conditions (i.e., with no control intervention whatsoever).

The most important variables for the models, despite the small differences, were precipitation and the number of eggs in the weeks prior to field collections. Figs. 4, 5, and 6 of the Supplementary Material show the importance of each predictor (variable) for Models 1, 2, and 3, respectively.

The impact of the control activities are shown in Table 3. The larval habitat control (CC) decreased the number of eggs collected by 43.56% (t + 1) in relation to what was predicted by the model. In the following two weeks, the larval habitat control maintained the effect of reducing the number of eggs collected in relation to what was predicted by the model: (t + 2: − 44.21% and t + 3: −52.79%). Similarly, adult control by insecticide nebulization also reduced the number of eggs collected in relation to what was predicted by the modeling (t + 1: − 79.82%; t + 2: − 43.41%; t + 3: − 61.83%) as well as the two types of treatment together (CC + NEB) also showed the effects on the number of eggs collected.

**Table 3:** Impact of interventions to control *Aedes aegypti.*

	CC (t + 1)	CC (t + 2)	CC (t + 3)	CC + NEB (t + 1)	CC + NEB (t + 2)	CC + NEB (t + 3)	NEB (t + 1)	NEB (t + 2)	NEB (t + 3)
count	85	85	85	69	69	69	31	31	31
mean (%)	−43.56	−44.21	−52.79	−68.72	−55.59	−55.10	−79.82	−43.41	−61.83
std	44.25	42.09	45.91	33.60	44.76	45.23	35.37	47.49	41.64
25%	−100.0	−95.22	−100.0	−100.0	−100.0	−100.0	−100.0	−92.49	−100.0
50%	−25.55	−43.65	−47.64	−81.20	−79.15	−75.02	−100.0	−9.96	−52.59

The results indicate that egg collections decreased by at least 25% of the sampled traps except for CC and NEB at t + 2. There was a 100% reduction in the number of eggs collected in relation to the values predicted by the mathematical model. No eggs were found in 25% of the traps—this was a 100% reduction relative to the value predicted by the models (with no intervention). In 50% of the traps, the reduction in the egg collections varied over all three types of control intervention. The best result was observed in areas with the combined effects of the two control activities (CC + NEB). The reduction in the number of eggs was maintained over three weeks after application of insecticide. There was a reduction by approximately 75% for eggs collected in 50% of the traps compared to the model's prediction. The effects of NEB intervention alone caused the highest decrease in the number of collected eggs versus what was predicted. The reduction in the number of eggs was 79.82% in the week following the treatment (t + 1). There was a 100% reduction in 25% and 50% of the traps. This effect remained at t + 2 and t + 3 with a significant reduction in the 25th and 50th quartiles. The CC is the only treatment that showed a progressive improvement over time in terms of overall effects on the number of eggs collected. A similar result was found when analyzing the reduction in the number of eggs collected in 50% of the traps (reductions of 25.55% (t + 1), 43.65% (t + 2), and 47.64% (t + 3)).

## 5 Discussion

The domestic form of *Ae. aegypti* is well-adapted to using artificial habitats for reproduction [7]. The major oviposition habitats of the domestic form are man-made recipients of several sizes and material [9,47]. Considering the adaptation of the species and its impacts on public health, monitoring its infestation in urban landscapes is demanding and important [48]. In addition, knowledge on the biology, ecology, and behavior of *Ae. aegypti* is necessary to tailor interventions and monitor reductions in mosquito population density [49].

The use of ovitraps as a tool in mosquito surveillance has advantages because they have a low implementation cost with less investment in training field personnel. This allows for allocation of financial resources to both testing and treating patients with arboviruses. Considering this premise and the sensitivity of the ovitraps in mosquito egg sampling, this study employed two analytical approaches to investigate the impact of the control activities—particularly from *Ae. aegypti* [33,34,50]. Field collections using ovitraps helped verify the dynamics of the mosquito oviposition weekly following the implementation of control measures.

Spatial analysis was used to verify the spatial correlation between control activities and their respective effects on the female population using the number of eggs found in the ovitraps as a proxy. The mosquito control measures adopted (CC, NEB and CC + NEB) presented negative spatial correlations with the number of eggs collected. In other words, there was a trend towards a smaller number of eggs collected in places where control actions occurred more frequently. The spatial maps generated in this study clearly show that the control activities displaced *Ae. aegypti* from the most critically infested areas in both space and time.

The superposition of this mapping information and correlation with the control activities in each town block produced asymmetric histograms. They showed that the distribution of the number of eggs collected in the ovitraps was smaller in blocks exposed to control than areas not exposed to any control. The control of *Ae. Aegypti* is a complex activity [51,52] that requires targeted actions involving entomological, health, economic, and social approaches. The anthropophilic behavior of *Ae. aegypti* requires strong participation of the communities and robust public policies aimed at management, for example, from recyclable materials that can represent a source of income for the population [47]. *Ae. aegypti* control does not depend on complex strategies, but rather on the use of already existing and largely tested methods with a combination of effective techniques and a constant sharing of information between scientific findings and vector control programs [49].

Similar results were also found using mathematical modeling. The treatments using CC, NEB, and CC + NEB caused a significant reduction in the number of eggs collected versus the number predicted by the mathematical model. Only the activities carried out to control the larval habitats produced a reduction of 43.56% at t + 1, 44.21% at t + 2, and 52.79% at t + 3. This type of control showed a progressive reduction in the number of eggs collected in the ovitraps during the three weeks. Controlling breeding sites is one of the most costly and difficult activities to control *Ae. aegypti* because the best results require a significant removal of all potential habitats [53]. The absence of residents in the homes—as well as the refusal of owners to allow control personnel to search for and remove all larval habitats from their household—are major challenges that must be overcome by the vector control program. CC can lead to an increase in the number of eggs collected in ovitraps due to the partial removal of available breeding sites in an area [54], but that was not seen here. The CC reduced the number of eggs collected in relation to what was predicted by the mathematical modeling in the three weeks following.

NEB had the greatest impact at t + 1 with a 79.82% reduction in eggs relative to the models' prediction. The NEB has an immediate impact on adult populations of *Ae. aegypti*, thus reducing potential risks of transmission of pathogens by mosquitoes in the area and influencing the dynamics of eggs collected in the weeks following control [55,56] as occurred here. The main advantage of this method is its fast implementation, but it is also environmentally destructive because it requires the application of non-selective insecticides in domestic environments [51].

The use of both CC + NEB showed the best cumulative result in the analysis of the quartiles. In 25% of the traps, this treatment reduced egg counts by 100% versus the values predicted by the mathematical modeling. In the 50th quartile, the association of CC + NEB reduced the number of eggs by 81.20%, 79.15%, and 75.02% in the first, second, and third week following treatment, respectively. There is a need to monitor the control methods used in day-to-day activities to deal with the existing vectors in each municipality because the effects may be different in each location, and the strategy for coping depends on these results to achieve their goals. The use of ovitraps is an effective way to monitor *Ae. aegypti* in the urban area [50]. This method was previously applied and there was spatial correlation between recyclers and *Ae. aegypti* infestation in urban areas [47]. Our results also indicate that ovitraps can be continuously employed to verify the effectiveness of vector control actions. The development of a mathematical model to verify the effect associated with the adequate installation of ovitraps allows for a better understanding of the results of each control approach. In the future, the most suitable period for each type of activity can be defined.

Limitations in financial and human resources are one of the biggest challenges in controlling *Ae. aegypti*. In this sense, our results demonstrate that the method of monitoring urban infestation through ovitraps along with control through fogging and removal of breeding sites can maximize available resources and produce an impact. Chemical control always results in environmental risk and human health, but the risks can be controlled if the methods used meet the technical recommendations. The products used in the control should be scientifically adequate. The joint application of CC + NEB treatments is the most efficient way to reduce *Ae. aegypti* infestation [51], but NEB alone can be an initial coping strategy immediately followed by CC. It is important to emphasize this issue because CC is more expensive and slower than NEB because it requires home visits with active search for breeding sites. This is expensive and time-consuming.

Machine learning can evaluate the routine activities of the vector control programs. The ovitraps require few financial and human resources. Likewise, the development of mathematical models can optimize actions to eliminate arboviruses. This is important for public health authorities because as it offers a very important tool for dealing with the vector and evaluating control strategies. This provides a more accurate decision-making intervention regarding what type of action will be used to control mosquitoes, thus minimizing the risk of outbreaks and epidemics and allowing one to establish the time that this particular type of control maintains its effect in the area affected by arboviruses.

## 6 Conclusions

Our results demonstrate that new spatial analysis tools and mathematical models are extremely important methods in vector control activities. They can lead to integrated solutions to problems that impact public health. The implementation of a monitoring system with ovitraps is sensitive and agile and can be used in areas with few resources to control Dengue. This type of trap is associated with the development of spatial and predictive models, and it allows one to evaluate control methods, thus aiding decision-making by health authorities and reducing health risks while improving the use of public resources.

## Authors' contributions

RP, TSA, and CJVZ conceived of the study. RP was responsible for all field collections and species identification. TSA and RP conducted all statistical and spatial analyses. FJVZ, RV, and ES analyzed the predictive mathematical modeling. RP, TSA, CJVZ, FJVZ, RV, ES and MAMS wrote the manuscript. All authors contributed to the final draft of the manuscript. All authors read and approved the final manuscript.

## Sources of funding

MAMS is financially supported by Conselho Nacional de Pesquisa—CNPq nº 301,877/2016–5; RV is financially supported by 10.13039/501100001807Fundação de Amparo à Pesquisa do Estado de São Paulo – FAPESP nº 2017/21174–8.

## Declaration of Competing Interest

The authors declare that they have no competing interests.

## Appendix A Supplementary data

Supplementary material Image 1
