# Supplementary material for: Assessing the effect of Aedes (Stegomyia) aegypti (Linnaeus, 1762) control based on machine learning for predicting the spatiotemporal distribution of eggs in ovitraps
Source: Dialogues Health. 2022 Feb 9;1:100003. doi: 10.1016/j.dialog.2022.100003 (PMC10954012; doi:10.1016/j.dialog.2022.100003)
Supplement: Supplementary file 1 — Supplementary material [file mmc1.docx]

**Supplementary material**

**Title:** Assessing the effect of *Aedes* (*Stegomyia*) *aegypti* (Linnaeus, 1762) control based on machine learning for predicting the spatiotemporal distribution of eggs in ovitraps

**Authors:**

Rafael Piovezan^1,2*^, Thiago Salomão de Azevedo^1,2^, Euler Sousa^3^, Rosana Veroneze^3^, Claudio José Von Zuben^2^, Fernando José Von Zuben^3^, Maria Anice Mureb Sallum^1^

**Affiliations**

^1^ Universidade de São Paulo, Faculdade de Saúde Pública, Departamento de Epidemiologia - São Paulo – SP, Brazil

^2^ Universidade Estadual Paulista, Departamento de Zoologia – Rio Claro – SP, Brazil

^3^ Universidade Estadual de Campinas, Departamento de Engenharia da Computação e Automação Industrial – Campinas – SP, Brazil

^*^Corresponding author: piovezan.rafael@gmail.com

**Materials and methods**

**Raw datasets**

Three datasets were made available, (1) collection of eggs, (2) control events and (3) rainfall index, with data ranging from January 2014 to February 2017. All the information was manually annotated by the health professionals of Santa Barbara d’Oeste. There was no key (unique row identifier) to join the information of these three datasets into a single dataset that could describe the time series of all variables of interest for each location. Therefore, a series of procedures was developed to produce a single dataset for later analyses. Those procedures are as follows.

The collection of eggs dataset is composed of a vast number of discrete events with non-constant time intervals. This characteristic is due to the sporadic pattern of collecting the eggs. An example of this dataset is presented in Table 1.

**Table 1** - Example of the egg collection dataset

| **year** | **season** | **month** | **week** | **date** | **address** | **lat** | **lon** | **location** | **Number of habitats number** | **Number of eggs** |
| --- | --- | --- | --- | --- | --- | --- | --- | --- | --- | --- |
| **2014** | summer | 1 | 1 | 2014-01-01 | Rua São Luis, 715 | -22.734327 | -47.385108 | 500.0 | 7 | 56 |
| **...** | ... | ... | ... | ... | ... | ... | ... | ... | ... | ... |

Nonetheless, a full history of these events can be sampled from the dataset for each location. We pre-processed this data aiming to achieve this goal by producing a weekly time series for each location in the city, taking into account the total number of *Ae.* *aegypti* eggs collected for that week and location. Table 2 displays an example of the egg collection dataset after treatment. Any week for a speciﬁc location that did not have any larval counting was ﬂagged as -1, representing a missing value, for further analyses.

**Table 2** - Example of the egg collection dataset after treatment

| **year** | **week** | **location** | **eggs** |
| --- | --- | --- | --- |
| **2014** | 1 | 500 | 56 |
| **...** | ... | ... | ... |

The original daily rainfall index dataset was used to calculate the average and standard deviation of the rainfall index for each week of the year, as presented in Table 3.

**Table 3** - Example of the rainfall index dataset after treatment

| **year** | **week** | **rainfall avg** | **rainfall std** |
| --- | --- | --- | --- |
| **2014** | 1 | 23.5 | 51.2 |
| **...** | ... | ... | ... |

The original dataset of control interventions has a similar pattern to the original egg collection dataset, both are composed of discrete events with non-constant time intervals. The characteristics of each type of control strategy, Nebulization (NEB) and Control of breeding locations (CC), are described in the Vector Control Section. Again, routines to build a weekly time series for each location were developed. An example of the dataset obtained is presented in Table 4, where the value 1 in the NEB or CC columns indicates that the control strategy was applied, and the value 0 indicates that the control strategy was not applied.

**Table 4** - Example of the control intervention dataset after treatment

| **year** | **week** | **location** | **NEB** | **CC** |
| --- | --- | --- | --- | --- |
| **2014** | 1 | 500 | 0 | 1 |
| **...** | ... | ... | ... | ... |

After each of the three original datasets was pre-processed in isolation, the resulting information was compiled into a single dataset using as key the combination of the following columns: year, week, and location. The ﬁnal dataset for the 1976 locations from 2014 to February of 2017 resulted in 322,088 rows and 8 columns, as displayed in Table 5.

**Table 5** - Example of the final dataset

| **year** | **week** | **location** | **eggs** | **NEB** | **CC** | **rainfall avg** | **rainfall std** |
| --- | --- | --- | --- | --- | --- | --- | --- |
| **2014** | 1 | 500 | 56 | 0 | 1 | 23.5 | 51.2 |
| **...** | ... | ... | ... | ... | ... | ... | ... |

**Training dataset**

After producing a single dataset with all necessary information, as exemplified in Table 5, a routine was developed to ﬁnd space-time promising regions with the following requirements:

• Time: 6-week time window.

• Region: 250 meters radius around each location (i.e., pair of latitude and longitude) available in the dataset.

• No intervention: only the time windows in which a control strategy intervention did not take place were considered.

This routine aimed to ﬁnd space-time promising regions, displaying the evolution pattern of the *Ae. aegypti* eggs over time in normal conditions. This routine yielded a total of 1,748 samples. Figure 1 exhibits a space-time promising region randomly selected from this resulting set of samples.

_
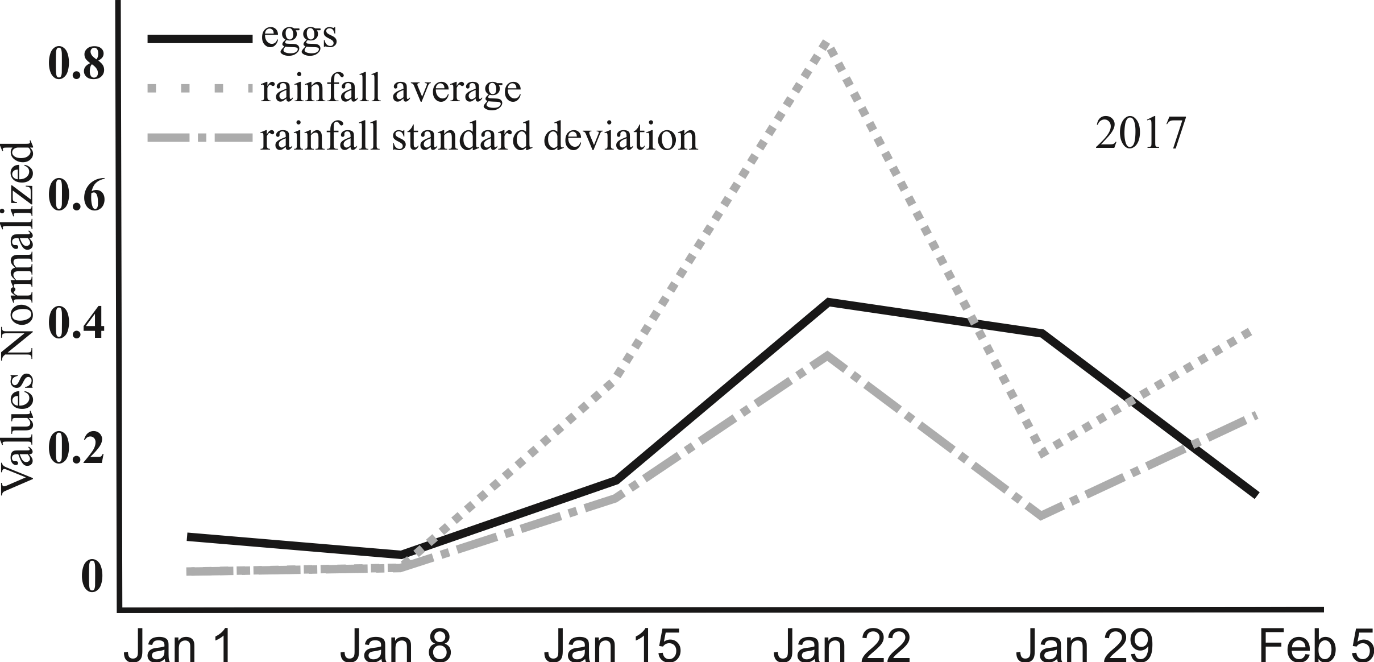
_

**Figure 1** - Example of a space-time promising region

These samples were used to train three non-linear autoregressive with exogenous entries (NARX) machine learning models with the goal of forecasting the number of *Ae. aegypti* eggs three weeks ahead: t+1, t+2, t+3. The procedures used for training the models are described in the Proposed models Subsection and the results obtained are presented in the Results Section.

**Inference dataset**

As discussed in the Inferring the effect of an event using the causal impact Subsection, it is necessary to have samples of the intervention methods analyzed to measure their causal impact in the target variable. Therefore, it was developed another search algorithm that could ﬁnd samples of 6-week window with a control strategy intervention in the third week from regions composed by a radius of 250 meters around each location (see an example in Figure 2). This search algorithm was able to ﬁnd 185 samples, which were split in three scenarios:

• Scenario 1: 85 samples with only larval habitats control intervention in the third week.

• Scenario 2: 69 samples with larval habitats control and nebulization interventions in the third week.

• Scenario 3: 31 samples with only nebulization intervention in the third week.


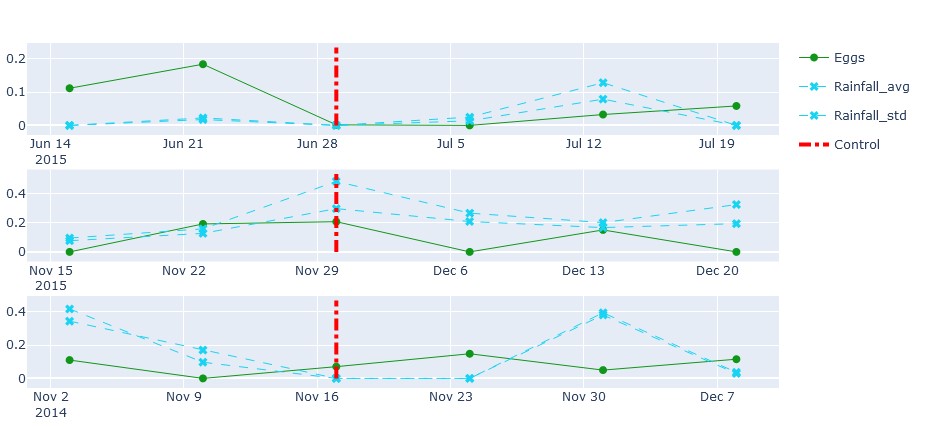


**Figure 2** - Example of 3 samples from the Inference dataset

**Proposed models**

We used three non-linear autoregressive with exogenous entries models for forecasting the number of eggs collected in the next three weeks, configuring a direct multi-step forecasting strategy. Each model was responsible for forecasting one week ahead, t+1, t+2 e t+3. The machine learning model selected for addressing this task was the Extreme Gradient Boosting Machine (XGBoost) [1].

The predictors and the target variable assigned to each model are described in the list that follows.

- Model 1:

Predictors:

rainfall − mean(t−3), rainfall − std(t−3), eggs(t−3),

rainfall − mean(t−2), rainfall − std(t−2), eggs(t−2),

rainfall − mean(t−1), rainfall − std(t−1), eggs(t−1),

rainfall − mean(t), rainfall − std(t)

Target: eggs(t+1)

- Model 2:

Predictors:

rainfall − mean(t−3), rainfall − std(t−3), eggs(t−3),

rainfall − mean(t−2), rainfall − std(t−2), eggs(t−2),

rainfall − mean(t−1), rainfall − std(t−1), eggs(t−1),

rainfall − mean(t), rainfall − std(t)

rainfall − mean(t+1), rainfall − std(t+1)

Target: eggs(t+2)

- Model 3:

Predictors:

rainfall − mean(t−3), rainfall − std(t−3), eggs(t−3),

rainfall − mean(t−2), rainfall − std(t−2), eggs(t−2),

rainfall − mean(t−1), rainfall − std(t−1), eggs(t−1),

rainfall − mean(t), rainfall − std(t)

rainfall − mean(t+1), rainfall − std(t+1)

rainfall − mean(t+2), rainfall − std(t+2)

Target: eggs(t+3)

The whole data available, containing 1,748 samples, was ﬁrst normalized and then divided using hold out validation scheme, with 80% (1,427 samples) for the training set and 20% (357 samples) for the test dataset. The training set was used for training the models and optimizing their hyper-parameters using the methodology explained below. The test dataset was left untouched and used only to evaluate the model performance.

For choosing the parameters of the models it was used a Bayesian Global Search Optimization (BSO) [2]. The boundaries of each parameter (i.e., the maximum and minimum values) was configured to limit the search space. Table 6 presents the search space deﬁned for the BSO process. For all other parameters, the default values were used.

**Table 6** - Search space of parameters

| **Parameter** | **Min** | **Max** |
| --- | --- | --- |
| **subsample** | 0.7 | 1 |
| **min*c_hild_w_eight_*** | 1 | 20 |
| **reg*l_ambda_*** | 1*e^−^*^4^ | 10 |

The BSO aimed at ﬁnding a local maximum (i.e., a minimum loss value) on the search space created by the distribution of values of each parameter respecting the minimum and maximum values conﬁgured.

At each BSO process iteration, 10-fold cross validation was performed, as shown in Figure 3.

_
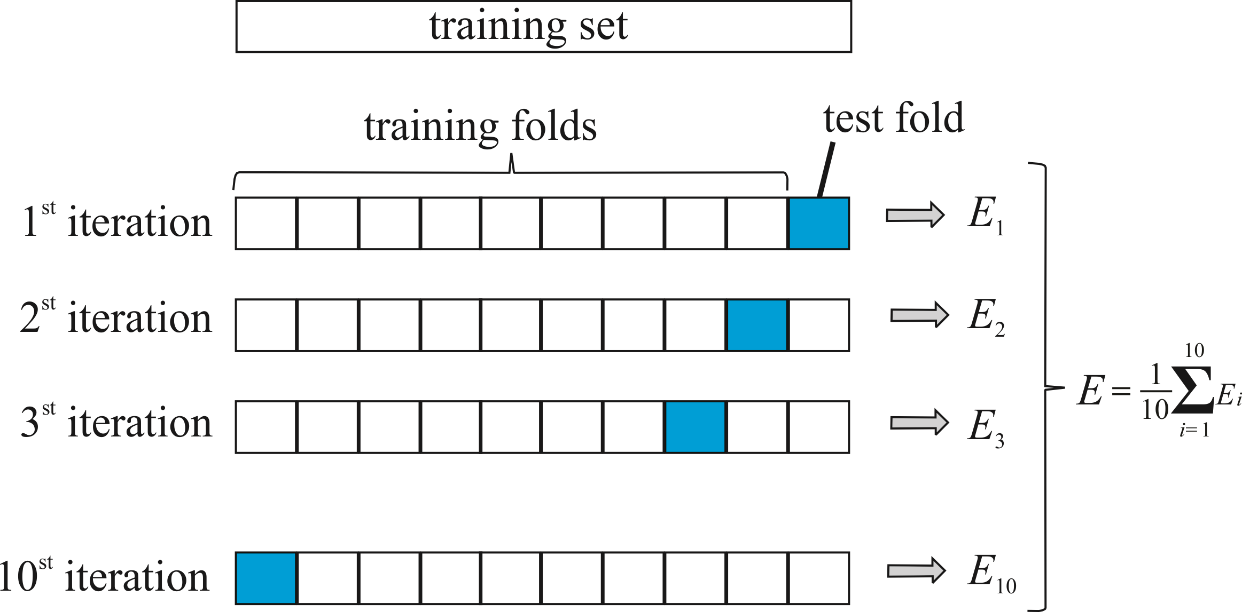
_

**Figure 3** - 10-folds cross-validation

The loss function selected for guiding the optimization process was the mean squared error (MSE):

__ (1)

where the index *j* represents the sample of the training fold ranging from 1 to *n*, *n* is the total number of samples in the training fold,
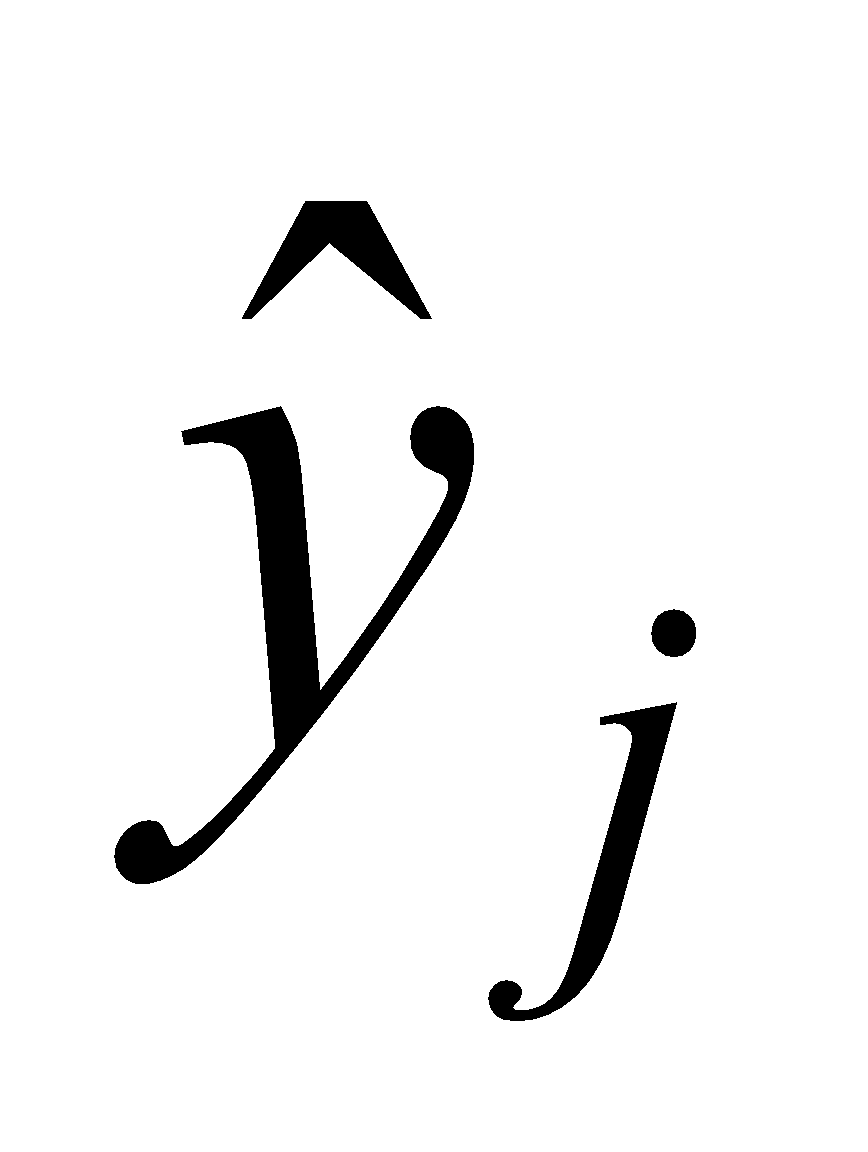
 is the predicted value,
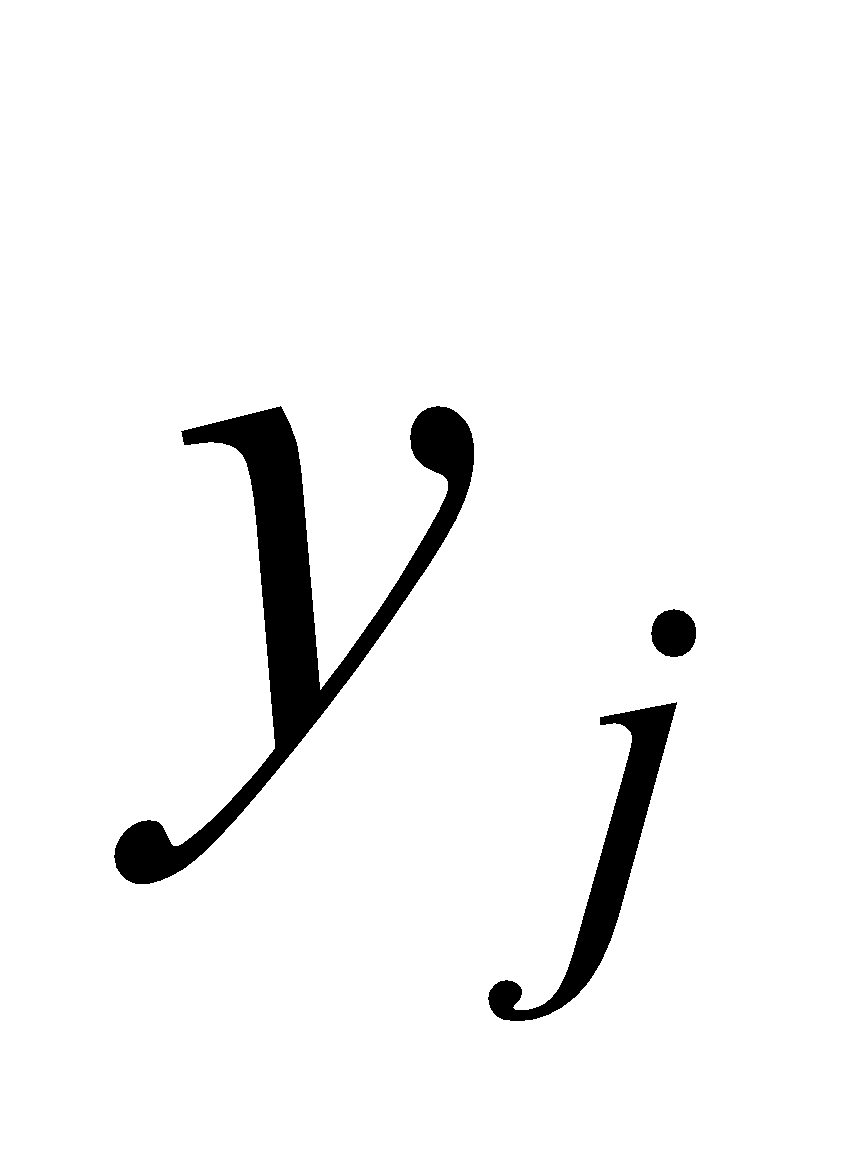
 is the true observed value, and the index *i* is the iteration of the *k*-fold.

As a result, each iteration of the BSO is a complete run of the *k*-fold scheme on the training dataset with the ﬁnal score *E* being the average MSE across all 10 folds, as presented in Equation 2. The standard deviation of the error is presented in Equation 3.

__ (2)

__ (3)

The main goal of the BSO process was to ﬁnd the best set of hyper parameters for each model that yields a minimum value of *E*. In other words, it was desirable to minimize the average of the MSE across all 10 folds of the k-fold cross-validation

**Results**

Figures 4, 5 and 6 show, respectively, the importance of each predictor (variable) for Models 1, 2 and 3.

**
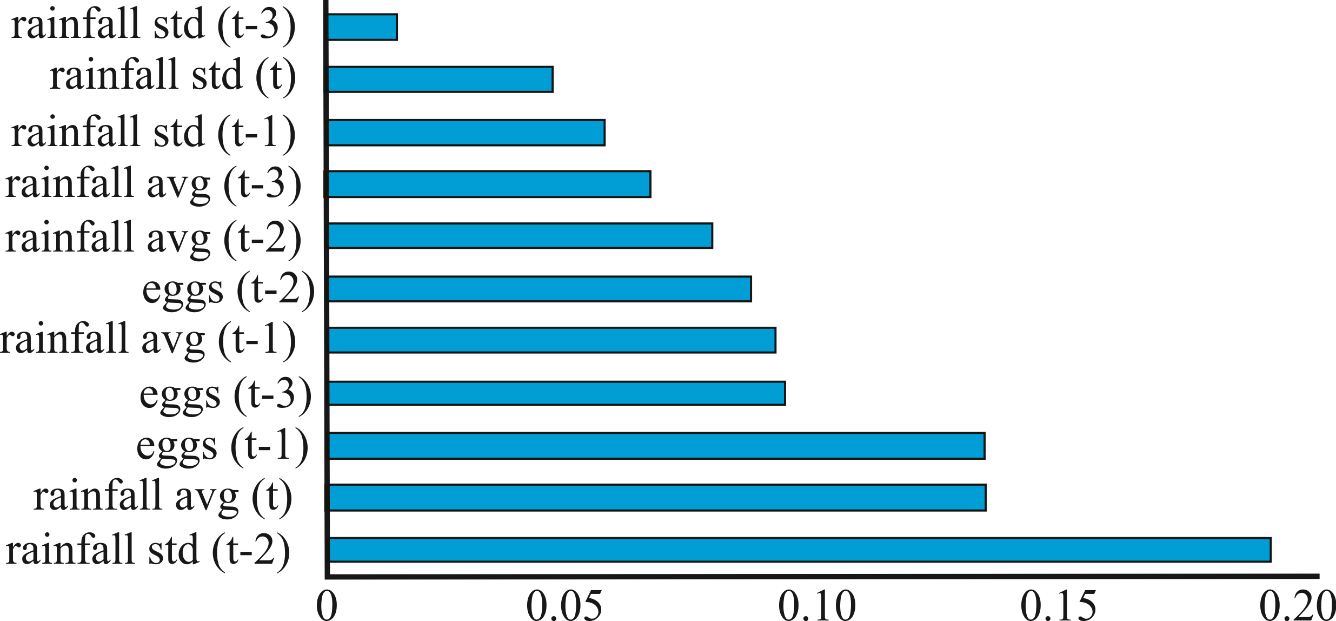
**

**Figure 4** - Importance of each predictor for Model 1

**
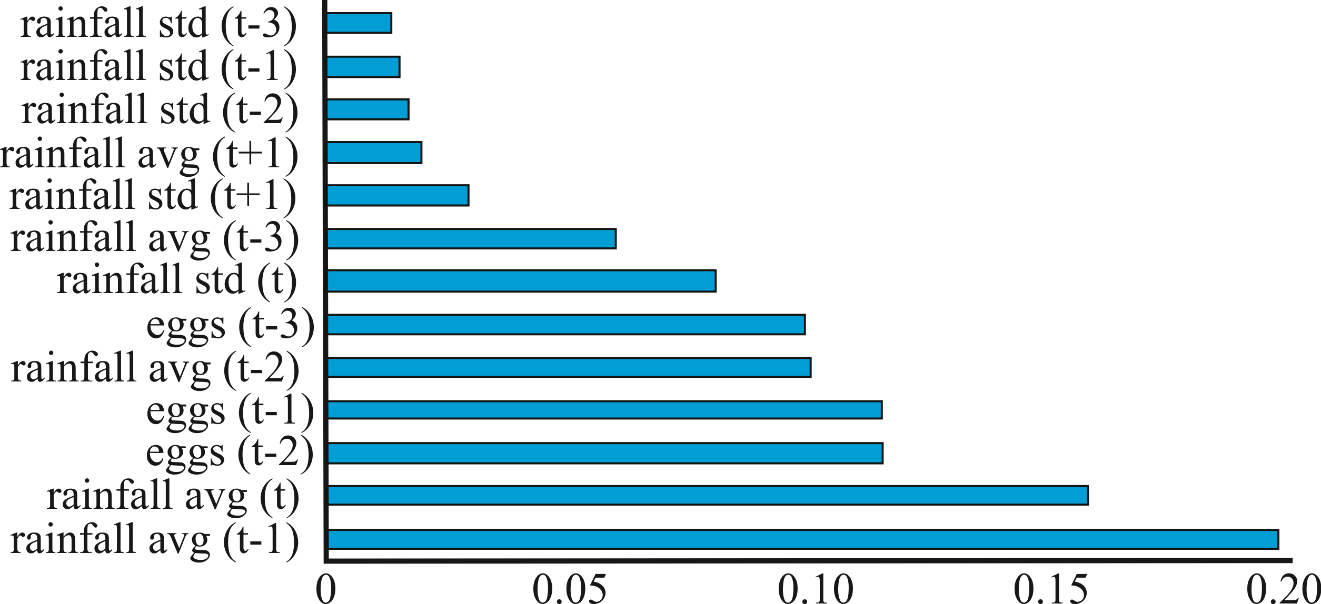
**

**Figure 5** - Importance of each predictor for Model 2

**
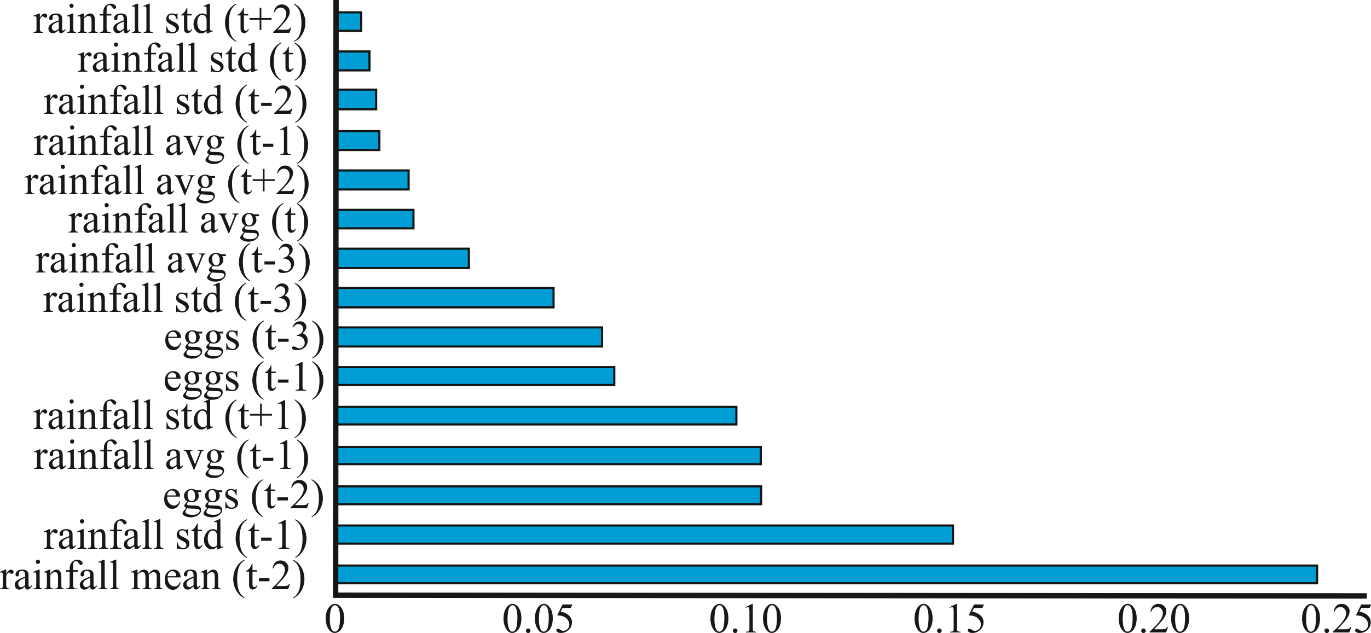
**

**Figure 6** - Importance of each predictor for Model 3

For each sample of the inference dataset, the trained models were used to predict the number of eggs in the timestamps t+1 (Model 1), t+2 (Model 2) and t+3 (Model 3). Figure 7 shows an example of the causal impact of an intervention CC+NEB. The counterfactual values are the predicted values.

**
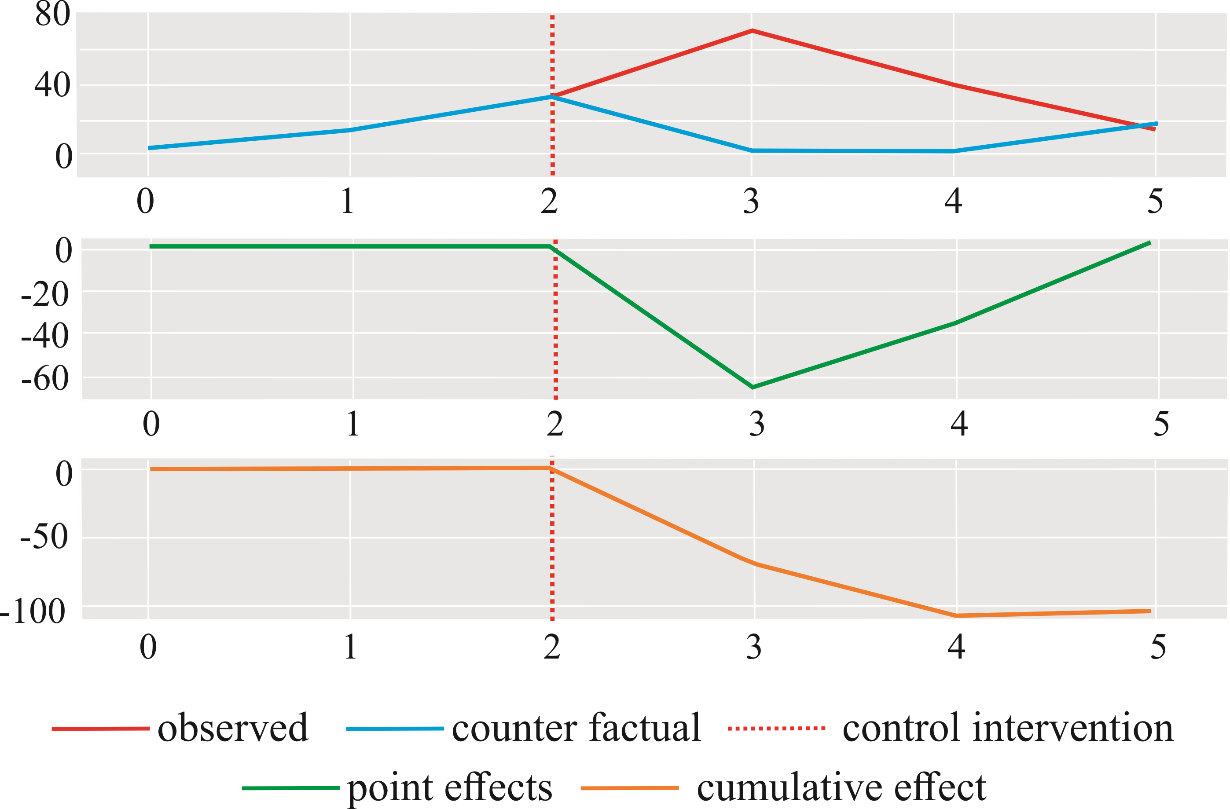
**

**Figure 7** - Example of the causal impact of an intervention CC+NEB

**References Supplementary Material**

[1] Chen T, Guestrin C. Xgboost: A scalable tree boosting system, CoRR, abs/1603.02754-2016, 2016.

[2] Nogueira F. Bayesian Optimization: Open source constrained global optimization tool for Python. URL https://github.com/fmfn/BayesianOptimization. 2014.
